# Multiparametric magnetic resonance imaging of the liver: bridging the gap between theory and practice - a bridge too far?

**DOI:** 10.1590/0100-3984.2021.54.5e1

**Published:** 2021

**Authors:** Ulysses S. Torres, Giuseppe D’Ippolito

**Affiliations:** 1 Grupo Fleury, São Paulo, SP, Brazil.; 2 Department of Diagnostic Imaging, Escola Paulista de Medicina da Universidade Federal de São Paulo (EPM-Unifesp), São Paulo, SP, Brazil. Email: giuseppe_dr@uol.com.br.

Chronic liver disease (CLD) is now a major public health problem worldwide, mainly due to the growing epidemic of obesity, alcohol consumption, and hepatitis. The central changes in the pathogenesis of CLD, such as fibrosis, necroinflammatory activity, iron overload, and fat accumulation, have traditionally been detected and monitored by biopsy, an invasive, costly, and limited procedure that is associated with non-negligible morbidity and mortality, making it imperative to replace liver biopsy with options that are more clinically practical, affordable, and accurate. Multiparametric magnetic resonance imaging (mpMRI) is currently one of the most promising tools for this task, because it makes it possible to use a wide range of advanced sequences that cover and analyze the complex spectrum of metabolic and cellular changes in the liver parenchyma, which has led some authors to consider a future in which virtual liver biopsy will be performed by mpMRI^(1)^. Preliminary evidence suggests that, in comparison with biopsy, mpMRI has a better cost-benefit ratio^(2)^.

Advanced MRI sequences for the evaluation of CLD include proton magnetic resonance spectroscopy (^1^H-MRS); proton density fat fraction measurement; T2 and T2* mapping; T1 mapping; elastography; diffusion-weighted imaging; susceptibility-weighted imaging; and dynamic series of temporal enhancement by paramagnetic contrast agents, to calculate the rate of intracellular uptake by hepatocytes and the extracellular volume fraction. Most studies of CLD have employed such sequences in different combinations to create a multiparametric protocol.

Currently, T1 mapping is a key component of multiparametric protocols for liver assessment, because the T1 relaxation time increases in parallel with an increase in the volume of extracellular fluid, which is characteristic of fibrosis and inflammation^(3)^. Given that the presence of iron (which can be accurately measured by T2* mapping) has the opposite effect on T1 relaxation time, some authors have developed an algorithm that adjusts for high iron content, generating corrected T1 (cT1) maps of the effects of iron^(3)^.Evidence suggests that cT1 mapping is more sensitive to subtle inflammatory changes in the liver than are measurement of serum biomarker levels and elastography^(4)^.

Among the various T1 mapping methods available, the modified look-locker inversion recovery sequence has garnered considerable attention because of its ability to generate high-resolution T1 maps during a single breath hold^(5)^. One obstacle to the wider use of cT1 mapping is that it is not available from major equipment manufacturers and is commercially available only as post-processing software for T2* mapping or proton density fat fraction measurement (LiverMultiScan; Perspectum Diagnostics, Oxford, UK), which makes it less accessible^(4)^.

In a seminal article on mpMRI, published in 2014, Banerjee et al.^(6)^ used a set of sequences consisting of T1 mapping, ^1^H-MRS, and T2 mapping to quantify liver fibrosis, steatosis, and hemosiderosis, respectively, showing that the performance of the method was excellent, with areas under the receiver operating characteristic curve > 0.9, for all three variables. Another study, published in 2017 and involving patients with nonalcoholic fatty liver disease, evaluated cT1 and T2* mapping, developing an imaging score for fibrosis and inflammation that was found to correlate significantly with the biopsy result^(3)^. Since then, liver MRI has come to be used ever more widely in a broad range of contexts, such as in the monitoring of patients with morbid obesity and nonalcoholic steatohepatitis (NASH) before and after bariatric surgery, as well as in clinical trials of treatments for NASH^(4)^; monitoring of nonalcoholic fatty liver disease in pediatric patients with overweight and obesity^(4)^; monitoring of the short-term response to treatment with direct-acting antivirals in patients with chronic hepatitis C^(7)^; and noninvasive monitoring and prediction of disease activity in children and adults under treatment for autoimmune hepatitis^(8,9)^.

Other studies have focused on the development of mathematical scores based on the combination of uncorrected T1 mapping, ^1^H-MRS, and magnetic resonance elastography for the prediction of NASH^(10)^, as well as T1 mapping based on the modified look-locker inversion recovery sequence before and after administration of gadoxetic acid (Primovist; Bayer Healthcare, Berlin, Germany), as a quantitative tool for estimating liver function and predicting esophageal or gastric varices^(5)^. There is a need for more multicenter clinical trials, in order to consolidate parameters, establish reference values, and promote the broader validation of mpMRI protocols in the evaluation of CLD from different causes in adults and children. In addition, which combination of sequences, if any, will be the most clinically efficient in predicting structural liver damage remains unknown.

In conclusion, there have been and will continue to be, in the coming years, an increasing number of exciting new developments the field of mpMRI, with technical refinements of sequences that are expected to be increasingly more accurate in detecting the gamut of parenchymal alterations in diffuse liver disease, thus avoiding the need for histological evaluation in many situations. In the context of the development and maturation of new treatments, the increasing calls for closer, noninvasive clinical monitoring of patients, and the low specificity of serum biomarkers, mpMRI appears to have enormous potential to fill an unmet need for better diagnostic tools and to transform clinical practice. The design and clinical validation of future imaging studies must take into account the challenge posed by the fact that percutaneous liver biopsy is not an ideal reference standard for fibrosis, steatosis, or hemosiderosis^(6)^, as well as the fact that it may be desirable to correlate the imaging findings with clinically relevant outcomes.
